# Supplementary material for: Young KRAB-zinc finger gene clusters are highly dynamic incubators of ERV-driven genetic heterogeneity in mice
Source: bioRxiv. 2025 Mar 2:2025.02.26.640358. Preprint. [Version 1] doi: 10.1101/2025.02.26.640358 (PMC11952569; doi:10.1101/2025.02.26.640358)
Supplement: Supplement 5 [file NIHPP2025.02.26.640358v1-supplement-5.pdf]

bioRxiv preprint doi: https://doi.org/10.1101/2025.02.26.640358; this version posted March 2, 2025. The copyright holder for this preprint (which was not certified by peer review) is the author/funder. This article is a US Government work. It is not subject to copyright under 17 USC 105 and is also made available for use under a CC0 license.

Table descriptions

Supplementary Table 1
Curated annotation of KZFP genes in the Chr4 cluster in BL6J, 129S1 and CAST mouse strains.

Supplementary Table 2
Zinc fingerprint arrays of coding KZFP genes in the Chr4 cluster in BL6J, 129S1 and CAST mouse strains.

Supplementary Table 3
TE content and enrichment at KZFP gene clusters

Supplementary Table 4
Percentage of divergence of LTR elements in BL6J, 129S1 and CAST strains, and *Mus spretus*

Supplementary Table 5
Summary of BL6J Chr4cl KZFPs' binding preferences: target TEs and sequence motifs.
